# Efficient Chemical Surface Modification Protocol on SiO_2_ Transducers Applied to MMP9 Biosensing

**DOI:** 10.3390/s21238156

**Published:** 2021-12-06

**Authors:** Ana L. Hernandez, Sidharam P. Pujari, María F. Laguna, Beatriz Santamaría, Han Zuilhof, Miguel Holgado

**Affiliations:** 1 Centre for Biomedical Technology, Universidad Politécnica de Madrid, Campus de Montegancedo, Pozuelo de Alarcon, 28223 Madrid, Spain; mariafe.laguna@upm.es (M.F.L.); beatriz.santamaria@ctb.upm.es (B.S.); m.holgado@upm.es (M.H.); 2 Laboratory of Organic Chemistry, Wageningen University & Research, Stippeneng 4, WE 6708 Wageningen, The Netherlands; sidharam.pujari@wur.nl (S.P.P.); han.zuilhof@wur.nl (H.Z.); 3 Department of Applied Physics, Escuela Superior de Ingenieros Industriales, Universidad Politécnica de Madrid, C/Jose Gutierrez Abascal, 28006 Madrid, Spain; 4 Department of Chemical, Mechanical and Industrial Design Engineering, ETS de Ingeniería y Diseño Industrial, Universidad Politécnica de Madrid, Ronda de Valencia 3, 28012 Madrid, Spain; 5 Institute for Molecular Design and Synthesis, School of Pharmaceutical Science & Technology, Tianjin University, 92 Weijin Road Nankai District, Tianjin 300072, China; 6 Department of Chemical and Materials Engineering, Faculty of Engineering, King Abdulaziz University, Jeddah 21589, Saudi Arabia

**Keywords:** surface modification, biofunctionalization, surface characterization, label free biosensor

## Abstract

The bioreceptor immobilization process (biofunctionalization) turns to be one of the bottlenecks when developing a competent and high sensitivity label-free biosensor. Classical approaches seem to be effective but not efficient. Although biosensing capacities are shown in many cases, the performance of the biosensor is truncated by the inefficacious biofunctionalization protocol and the lack of reproducibility. In this work, we describe a unique biofunctionalization protocol based on chemical surface modification through silane chemistry on SiO_2_ optical sensing transducers. Even though silane chemistry is commonly used for sensing applications, here we present a different mode of operation, applying an unusual silane compound used for this purpose (3-Ethoxydimethylsilyl)propylamine, APDMS, able to create ordered monolayers, and minimizing fouling events. To endorse this protocol as a feasible method for biofunctionalization, we performed multiple surface characterization techniques after all the process steps: Contact angle (CA), X-ray photoelectron spectroscopy (XPS), ellipsometry, and fluorescence microscopy. Finally, to evidence the outputs from the SiO_2_ surface characterization, we used those SiO_2_ surfaces as optical transducers for the label-free biosensing of matrix metalloproteinase 9 (MMP9). We found and demonstrated that the originally designed protocol is reproducible, stable, and suitable for SiO2-based optical sensing transducers.

## 1. Introduction

Biosensors devices have grown as a technology to induce an unprecedented change in detection and monitoring systems for multiple healthcare applications [1,2]. Biosensors are devices that measure biological responses by generating a signal indicative of the concentration of the analyte in the studied sample [3]. They are typically composed of a bioreceptor attached to a transducer and a detection system to translate the transducing signal into understandable information. The transducing signal can have different natures such as magnetic [4], electrochemical [5,6], piezoelectric (mass detection methods) [7], micromechanical [8], thermal [9], or optical [10]. Among all types of biosensors, label-free biosensors’ operational simplicity has led them to be a potential tool to complement or even displace other detection technologies, especially for integrated and miniaturized point of care systems and lab-on-a-chip applications [11,12,13,14].

The process of immobilization of biological species onto the transducers (bioreceptors) for the specific target molecule binding is referred to as biofunctionalization and plays an essential role in achieving an effective and reliable label-free biosensor. Obtaining a reproducible, homogenous, and effective biofunctionalization protocol is crucial for the functioning of such biosensors [15]. This process needs to be adapted to the analytes of interest. Ideally, it should consist of only a few steps, covering the surface homogeneously in a compatible manner with the properties of the sensing surface. Biofunctionalization should avoid non-specific binding (fouling) [15] and minimize the reagent volumes involved. It is a critical phase that can affect the biosensors’ performance. Some unsuitable events can occur during the biofunctionalization process that must be avoided: physical or chemical damage of the biomolecule, affecting its conformation and affinity properties, non-specific binding, randomness in the orientation of the biomolecules, blocking off the reactive binding groups, inhomogeneity along the surface and matrix effects within the sample containing the bioreceptor. Very often, new biosensors being reported in the literature rely on general biofunctionalization approaches that are not specific to the type of biosensor or bioapplication being shown. They are based on general assumptions and experimental conditions, leading to a vague description of the biofunctionalization protocol, hardly reproducible.

In this work, we carefully describe a chemical-surface modification protocol on SiO_2_ surfaces for anti-MMP9 immobilization for optical label-free biosensing of MMP9. We meticulously describe the different steps of the process, from surface modification groups to the final detection of the analyte. We proof and provide a deep explanation of the different chemical reactions taking place along the process, and we describe the specific conditions of the different steps to facilitate the reproducibility of the protocol. For each of the steps, we report different characterization techniques, CA (Contact Angle), ellipsometry, XPS (X-ray photoelectron spectroscopy), fluorescence, that indicate the effectiveness of the described biofunctionalization process. Among the various approaches for label-free optical biosensing, including surface plasmon resonance devices, ring resonators, photonic crystals, and Mach–Zehnder devices [16,17,18,19], we used an interferometric optical biosensing based on vertical interrogation [20,21].

### Silane Based Chemistry

For planar silicon-based substrates, the use of alkoxysilanes provides a covalent link between the organic and SiO_2_ phase [22,23] that creates a homogeneous thin layer with a good surface coverage, robustness, low non-specific binding, minimal sample and reagents consumption, and easy handling [15]. The alkoxy groups in alkoxysilanes react with hydroxyl (OH) groups on activated silicon oxides, thus forming a covalent -Si-O-Si- bond [24], while the remainder of the molecule can bear a range of different functional groups to couple the bioreceptor. Examples include aminosilanes (NH_2_-organosilanes), glycidosilanes (epoxy-organosilanes), and mercaptosilanes (SH-organosilanes). Amine-terminated silanes are a good option for immune biosensors based on antibody–antigen-specific binding. Functional groups NH_2_ can couple to a carboxylic acid (COOH) from the heavy chain [25] of antibodies, yielding amide formation [26] and facilitating the antibodies conjugation in the desired orientation, where antigen-binding sites remain available for molecules recognition.

To demonstrate the benefits of the use of 3-(ethoxydimethylsilyl)propylamine (APDMS) [23] in this work rather than other very well-known and more commonly used aminosilanes, for example (3-aminopropyl) triethoxysilane APTES [27] or (3-aminopropyl) trimethoxysilane APTMS [28,29], we compared the monolayer formation of APDMS and APTMS. Regardless, it is widely used, and APTMS monolayer formations are reported within a wide range of concentrations, solvents, or reaction times and characterization results (e.g., seen in the variation of contact angles reported in the literature for this modified surface) [30]. Thus, the applied conditions for its use in the monolayer formations lack consistency, which results in hardly reproducible results. APTMS is composed of a silicon molecule bonded to 3 methoxy groups (that react to hydroxyl groups from the SiO_2_ surface) and to a propyl chain with one amine functional group with the nucleophilic property. Nevertheless, this silane rarely forms a perfect monolayer since it has a great tendency to polymerization due to the presence of three methoxy groups overreacting with amine groups of near molecules. This prevents the homogeneity along the surface and disturbs the availability of the amine functional group. This undermines the standardization protocol for its proper application.

In literature, we found APDMS (18306-79-1 Sigma Aldrich, Zwijndrecht, The Netherlands), which like APTMS (281778 Sigma Aldrich), has an amine functional group and a propyl chain bonded to a silicon molecule. However, it poses two methyl groups and a single ethoxy group, promising a more stable single monolayer formation. Therefore, we studied monolayer formation comparing both silanes before proceeding to biofunctionalization steps. APDMS showed higher consistency in the monolayer formation in terms of X and Y axes and thus was applied for the first time for anti-MMP9 immobilization on SiO_2_ surfaces.

We used this silane compound in this work to bring a new and effective path for silicon-based surfaces biofunctionalization as an element for effective optical label-free immunosensing purposes.

## 2. Materials and Methods

### 2.1. Monolayer Formation

**Figure 1:**
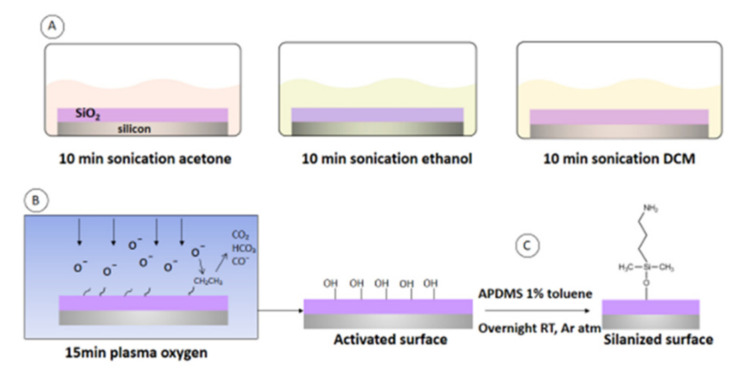
(**A**) Monolayer formation steps. Washing for 10 min in acetone, 10 min in ethanol, and 10 min in DCM. (**B**) 15 min plasma surface activation (creation of OH groups). (**C**) Silane (APDMS) reaction to -OH groups generating a silanized surface.

Hydroxyl-terminated surface preparation: 1 × 1 cm chips of 660 nm of silicon oxide on the silicon substrate were consecutively sonicated for 10 min in acetone, ethanol, and DCM (dichloromethane). Subsequently, the chips were dried by a stream of argon and placed into a plasma activation chamber (Diener electronic Plasma-Surface-Technology Femto). The activation chamber was pumped down to less than (1.2 ± 0.2) × 10^−2^ mbar prior to the introduction of oxygen plasma. Pieces of silicon-oxide substrates were placed in the plasma cleaner and oxidized for 15 min with 0.5 sccm, oxygen (>99%) flow (Q-Flow), 29.6 W power (100%), at 0.2 mbar pressure (Figure 1A,B).

Surface modification: We studied monolayer formation comparing both silanes before proceeding to the biofunctionalization steps. Dry toluene was added to the samples, followed by the silane to obtain a silane concentration of 1% (*v*/*v*) in the mixture. (Figure 1C). Then, the solution was stirred overnight in an argon atmosphere followed by sonication for 1 h to remove any polymerized silane. Next, it was dried with nitrogen and kept for 1 h in an oven at 110 °C to remove any unbounded molecules.

The same process proceeded for both silanes evaluated at this stage.

### 2.2. Biomolecules Immobilization

Anti-MMP9 (anti-mouse monoclonal antibody, MAA553Hu22 Cloud-Clone Corp.) was immobilized on the silanized surfaces after amine groups activation through a suitable linker. This process was performed only on samples modified with APDMS silane. Bovine Serum Albumin (BSA) (05470 Sigma Aldrich) was also immobilized on the silanized surface to prove the robustness of the silanization process.

#### 2.2.1. Amine Activation

After the monolayer formation, the functional amine group needs to be activated to allow the coupling of the antibody to the silanized surface.

There exist several activators commercially available that react with the amine group, such as disuccinimidyl carbonate (DSC), N-hydroxy succinimide (NHS), glutaraldehyde (GA) [29,31,32,33]. For this purpose, we used carbonyl diimidazole CDI (115533 Sigma Aldrich) since this chemical is easy to use in water solution, and successful results have been reported in the literature [34].

**Figure 2:**
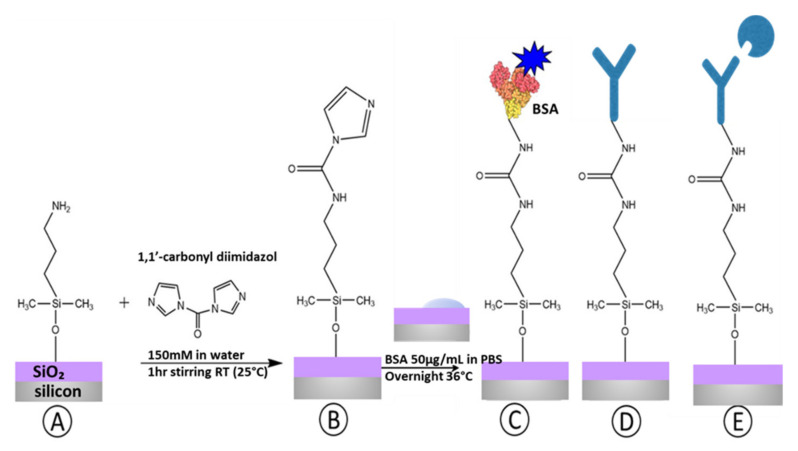
(**A**) Silanized surface, (**B**) NH_2_ activation through CDI coupling, (**C**) BSA biofunctionalization, (**D**) Anti-MMP9 biofunctionalization, (**E**) MMP9 recognition.

Modified surfaces were immersed in a solution of 150 mM of CDI in water and stirred for 1h at room temperature. (Figure 2B).

#### 2.2.2. Bovine Serum Albumin (BSA) Immobilization

A drop of 50 μg/mL BSA in phosphate buffer saline 10 mM (PBS) was incubated overnight at 36 °C in humid conditions on amine-activated surfaces. BSA was labeled with alexa fluorophore 488 to analyze biofunctionalization success under the fluorescence microscope. After incubation, the surface was energetically rinsed with PBS-t (Phosphate Buffer Saline solution with Tween detergent) and Di-water and dried with N_2_ gas. The drop of BSA did not cover the whole surface of the SiO_2_ chip, but only a specific area, in order to have a background free of BSA for the fluorescence analysis. (Figure 2C).

#### 2.2.3. Antibody Immobilization

For biosensing analyses, 50 μL of 50 μg/mL anti-MMP9 solution in PBS was incubated on the amine-activated surfaces overnight in humid conditions at 36 °C. (Figure 2D).

A polydimethylsiloxane (PDMS) mask was used, defining three independent circular sensing areas (1 mm diameter) in the same chip, named cell A, cell B, cell C. The purpose of this was to obtain three independent optical interferometers on the same chip to demonstrate the stability of the processes along the surface of the chip for future multiplexing biosensing applications.

For obtaining fluorescence images, labeled antibodies (anti-goat CF 488A, Sigma Aldrich) were also immobilized on a silanized SiO_2_ surface.

### 2.3. MMP9 Recognition

The MMP9 (RPA553Hu01 Cloud-Clone Corp) recognition (Figure 2E) was exclusively tested by optical interrogation to demonstrate that the surface modification and biofunctionalization protocol proposed is suitable for silicon-based optical biosensing transducers. On a biofunctionalized surface with anti-MMP9, 50 µL of ethanolamine 0.1 mM was incubated for 2 h at 37 °C, in humid conditions, to block the surface and prevent fouling processes. Then, consecutive increasing concentrations of MMP9 were incubated for 90 min and measured one after another to obtain the recognition curve. After each incubation process of biomolecules, samples were washed with Di-water and dried with clean oxygen before the optical interrogation.

### 2.4. Surface Characterization

- **Static Contact Angle (SCA):** Static water angle measurements were made with an automated Krüss DSA 100 goniometer. According to the size of the modified surfaces, 1–2 droplets were dispensed on the surface, and the contact angles were determined using a Tangent 2 fitting model. The standard error in the determined contact angles is approximately 1°. It was obtained from 5 different samples.
- **Ellipsometry:** The ellipsometry thicknesses of the samples were assessed by using a Sentech Instruments type SE-400 ellipsometer, operating at 623.8 nm (He-Ne laser), and an angle of incidence of 70°. The mode “polarizer+retarder, aperture, strict” was used. The optical constants of a freshly etched hydrogen-terminated Si(111) surface were taken as n = 3.821 and k = 0.057. The reported values are the result of a planar three-layered (ambient, monolayer+ silicon oxide and silicon substrate, see Appendix A, Figure A6) model with the assumed refractive indices of 1.00 and 1.46 for the ambient and monolayer + underlying silicon oxide, respectively. The thickness of the silicon oxide layer was measured separately on a plasma cleaned unmodified of the same wafer; it was 672 ± 0.2 nm. The final monolayer thickness was obtained by the subtraction of these separately measured silicon oxide layer thickness from monolayer+silicon oxide (see Appendix A, Figure A6). All the reported values are averages of at least 5 measurements, and the error is approximately 0.2 nm.
- **X-ray Photoelectron Spectroscopy (XPS):** X-ray photoelectron spectroscopy (XPS) spectra were obtained on a JPS-9200 photoelectron spectrometer (JEOL, Japan). The analysis was performed under ultra-high vacuum conditions using a monochromatic Al Kα X-rays (hν = 1486.7 eV) at 12 kV and 20 mA and an analyzer pass energy of 10 eV. A take-off angle φ of 80° was used. All the XPS spectra were processed with Casa XPS software (2.3.18), and the binding energies were calibrated on the hydrocarbon (CH_2_) peak with a binding energy of 285.0 eV.
- **Fluorescent Microscope:** Labeled BSA and labeled antibodies were characterized by a digital microscope (BX51, Olympus).

### 2.5. Optical Biosensing

The surfaces of SiO_2_ were used as Fabry–Perot optical interferometric transducers (interferometers) [35] for optical vertical interrogation for anti-MMP9 and MMP9 label-free detection.

Increasing concentrations of MMP9 were incubated on anti-MMP9 biofunctionalized surfaces blocked with ethanolamine. After every incubation, transducers were rinsed and dried with clean air before the optical biosensing.

**Figure 3:**
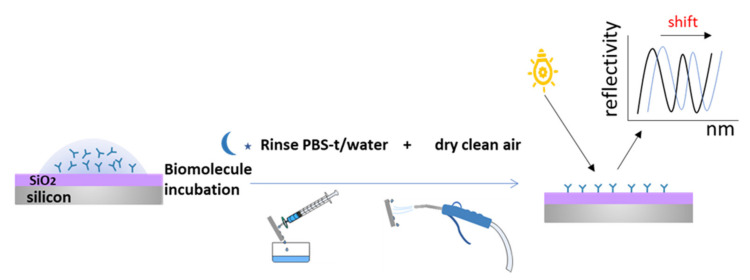
Incubation of molecules on surface, washing, drying, and optical vertical interrogation of the surface for biosensing results.

The biosensing principle is based on the identification of changes in the surface of the interferometer due to the refractive index variation caused by the immobilization processes (antibodies) and the recognition events (MMP9 detection). The interrogation system captures the interference signal, which is represented as an optical mode showing the reflectivity of the sample as a function of wavelength (nm). This optical mode shifts according to the biological material percentage immobilized on the studied interferometric surface. (Figure 3).

The interrogation system used was a high-resolution FT-VIS-NIR Spectrometer (BrukerVERTEX70); each measurement was taken 3 repeated times to estimate the uncertainty of the results. The measurements were performed with 200 scans, and the resolution of the system was 0.01 nm [36]. We used a 4x objective at a normal incidence for the interrogation of the 3 different independent interferometers of the transducer defined by the PDMS mask.

## 3. Results & Discussion

We studied the monolayer formation of the APTMS to compare it against less commonly used APDMS in order to justify the application of the selected silane over other commercial silanes. In Table 1, there are summarized results comparing APTMS and APDMS.

We obtained an SCA of 52° ± 1 and 55° ± 1 for APTMS and APDMS respectively, which suggest, in principle, higher hydrophilicity for APTMS. Hydrophilicity is a feature that is pursued when attempting to modify the surface of a biosensing transducer to enhance the bioreceptor immobilization. However, the thicknesses of APTMS drawn from ellipsometer and from XPS results are 4.3 ± 0.2 nm and 3.0 nm, respectively, which does not match the theoretical thickness of APTMS, which is around 1 nm. These results reveal a multilayer formation in APTMS modified samples. In contrast, APDMS results were 0.6 ± 0.2 nm and 0.5–0.8 nm thick for the respective methods mentioned.

The monolayer thickness was calculated from these XPS C/Si ratios using the following Equation (1):

(1) $$d_{ML}\left(Å\right)\;=\lambda_{ML}^{Si}\sin\left(\varphi \right)\;\ln\;(1+\frac{C}{Si})$$

with $\lambda_{ML}^{Si}$ = attenuation length of Si 2p photoelectrons in the organic monolayers ($\lambda_{ML}^{Si}=$ 39.5 Å), and *φ* = take-off angle between the surface and the detector (in this experiment: φ = 80°).

In addition, the C/N ratio is expected to be 3 for the surface treated with APTMS, but results from XPS showed a C/N of 8.5, from which it can be inferred that there is an extra proportion of carbon over nitrogen due to the polymerization of the molecules and thus, limiting amine groups availability. Unlike this, the C/N of APDMS (5.6) surface is within the expected C/N from the molecule structure, which is 5.

APTMS has three reactive alkoxy groups to bind OH from the activated surface. This can lead to polymerization where alkoxy groups bind NH_2_ functional groups from nearby silane molecules. On the contrary, APDMS only poses one alkoxy group, which facilitates the ordered union to the OH radical groups and a monolayer formation.

These results support the decision to continue the work using APDMS for surface modification and next biofunctionalization and biosensing experiments.

### 3.1. Ellipsometry (nm) and SCA of the Different Biofunctionalization Steps

An ellipsometer was used to determine the increased thickness after the different reported steps (monolayer formation with APDMS, CDI linker for amine activation, and biofunctionalization with label BSA and anti-MMP9) and the surface hydrophobicity was studied under the SCA (Table 2). A blank sample of SiO_2_ after O_2_ plasma treatment was measured as a reference, and thickness 0 and SCA 0° were obtained. SiO_2_ sample thickness after APDMS monolayer formation was 0.7 ± 0.2 nm. As expected, the result was under 1 nm, indicating the formation of an ordered monolayer and not the undesirable multilayer, which would hamper the biomolecule binding to the surface. In addition, the SCA was 55° ± 1°, which is in the range of SCA found in the literature for optimal silanized surfaces [37].

Monolayer formation is a crucial step. We repeated the measurement in 5 repeated silanization processes on different days to verify that the monolayer formation was reproducible and stable. Results shown in Table 2 are the arithmetic mean of those 5 repeated measurements.

After CDI conjugation, SCA decreased regarding the previous step of monolayer formation (from 55° ± 1° to 47° ± 1°) from what can be inferred as an increased hydrophily of the surface due to the amine activation. Also, total thickness increased to 0.8 ± 0.2 nm due to the CDI addition. After labeled BSA (~3 nm) incubation, thickness increased considerably, up to 3.14 ± 0.2 nm due to the addition of biological material. SCA also increased to 75° ± 3°, showing a more hydrophobic surface, which is expected for biofunctionalized surfaces with labeled molecules. Antibody incubation provoked an increased surface thickness of 16.7 ± 3 nm, which is close to the range expected, according to the antibodies’ size around 12–16 nm (ref). SCA, in this case, was 67° ± 3° suggesting that surface had lost its hydrophilicity after the antibody’s immobilization.

### 3.2. XPS (SiO_2_-APDMS-CDI-antiMMP9)

**Figure A1:**
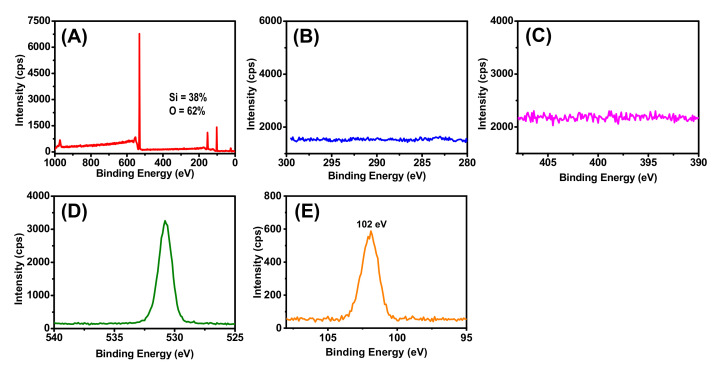
XPS analysis of 10 min plasma oxidized SiO_2_ surface. (**A**) wide, (**B**) C 1s, (**C**) N 1s, (**D**) O 1s, and (**E**) Si 2p spectra.

**Figure A2:**
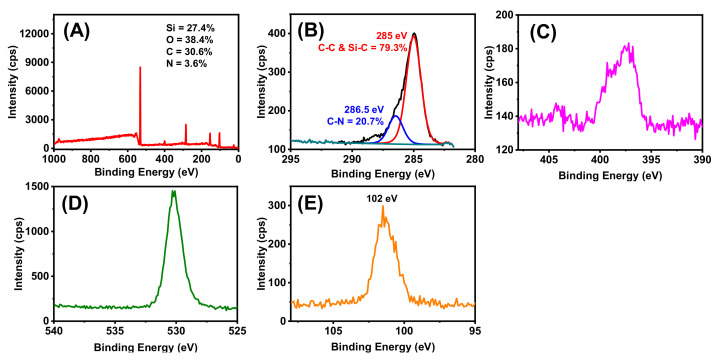
XPS analysis of APTMS on plasma cleaned SiO_2_ surface. (**A**) wide, (**B**) C 1s, (**C**) N 1s, (**D**) O 1s, and (**E**) Si 2p spectra.

**Figure A3:**
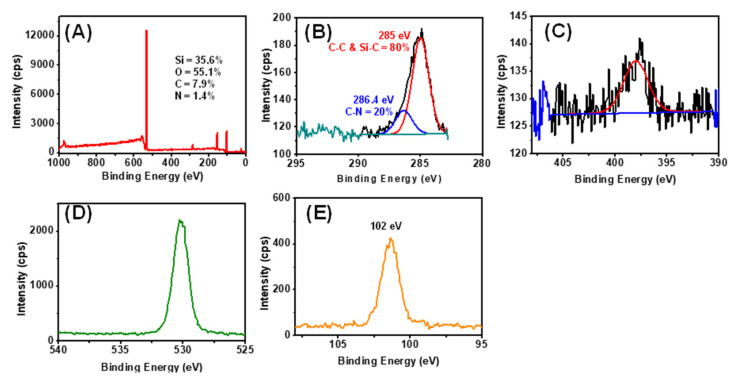
XPS analysis of APDMS on plasma cleaned SiO_2_ surface. (**A**) wide, (**B**) C 1s, (**C**) N 1s, (**D**) O 1s, and (**E**) Si 2p spectra.

Table 3 and Appendix A presents the results of XPS analyses of surfaces modified with a series of functionalization on the SiO_2_ surface. The stepwise procedure started with the plasma clean for the formation of the hydroxyl-termination on the SiO_2_ surface. Detailed characterization of the plasma cleaned SiO_2_ surface shows the absence of a carbon signal at 285 eV (Figure A1A in Appendix A), which is an ideal starting point to proceed to the surface modification with ultra-thin monolayers of APDMS and APTMS. The SiO_2_ cleaned surface was further modified with APDMS and APTMS separately. However, APTMS shows multilayer formation according to the element’s percentages found, which are 30.6 and 3.6 for carbon and nitrogen, respectively (Figure A2). On APDMS monolayers, it is 7.9 and 1.4 percentage of carbon and nitrogen, respectively (Figure A3). These results suggest that APDMS forms perfect single monolayers, which is in concordance with the ellipsometry thickness measurement.

**Figure A4:**
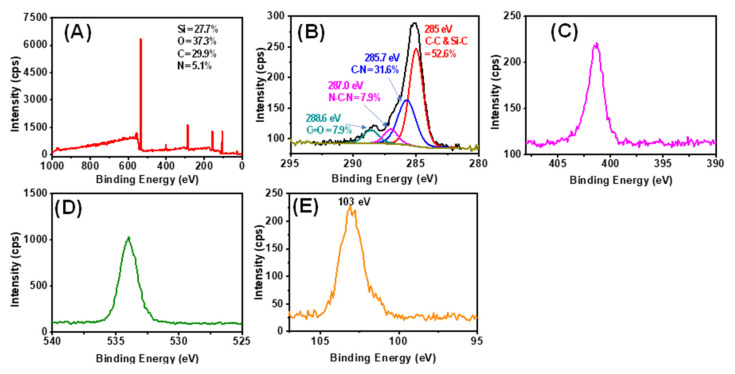
XPS analysis of CDI coupled APDMS-SiO_2_ surface. (**A**) wide, (**B**) C 1s, (**C**) N 1s, (**D**) O 1s, and (**E**) Si 2p spectra.

**Figure A5:**
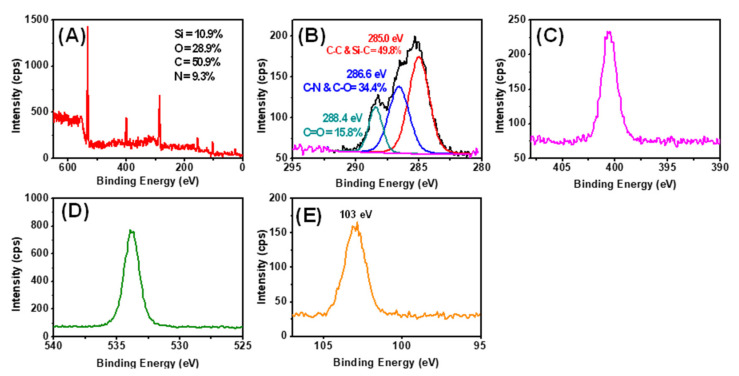
XPS analysis of antiMMP9 on CDI coupled APDMS Si(111) surface. (**A**) wide, (**B**) C 1s, (**C**) N 1s, (**D**) O 1s, and (**E**) Si 2p spectra.

**Figure A6:**
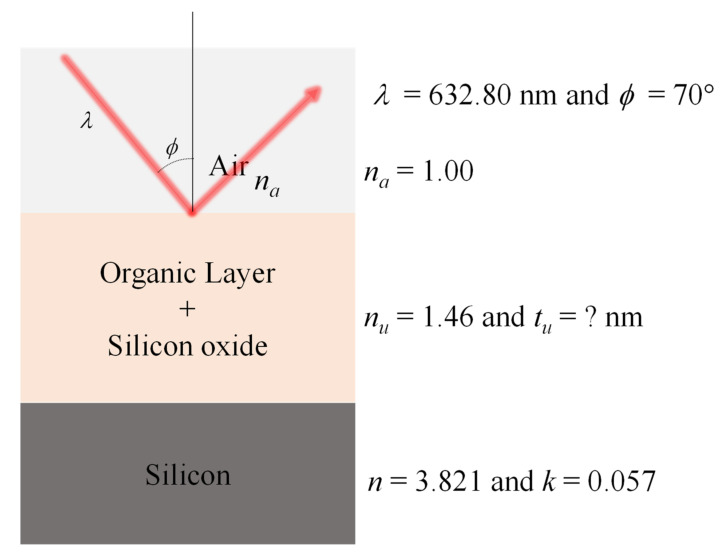
Model of the three layers used for the analysis of the ellipsometric results. Right side notations and numbers are dielectric constants were used to obtain ellipsometry thickness (tu). Whereas n_a_ = ambient air, n_u_ = upper organic layer + silicon oxide layer, t_u_ = thickness of upper organic layer + silicon oxide layer, and n = refractive index of silicon, k = extinction coefficient of silicon. In addition, plasma cleaned unmodified silicon oxide layer on silicon was measured 672 ± 0.2 nm.

The APDMS surface was further activated with CDI reagent (Figure A4). After that, XPS analysis, wide spectrum showed carbon and nitrogen percentages of 19.5 and 5.8, respectively. The relative C/N is in good agreement with the expected 9/3 molar ratio. The spectrum was decomposed into 3 main peaks, and each was assigned to the different carbons present in the attached APDMS and CDI monolayer (Figure A4B). In that figure, the C 1s signal at 285 eV corresponds to the methylene carbon atom involved in the Si-C and C-C groups; also, hydrocarbon CH_2_ C 1s is calibrated at this binding energy. The shoulder peak observed at 285.5 eV corresponds to the C-N, and the peak at 287.0 eV corresponds to the N-C-N from the CDI group. Finally, at 289.1 eV it is observed the C=O group. In addition, the active CDI surface was employed to immobilize anti-MMP9. In XPS, the spectrum for anti-MMP9 (Figure A5) shows a very high percentage of carbon due to the size of the biomolecule, which is almost 50.9%, as shown in Table 3.

### 3.3. Fluorescence (BSA and Labeled Antibodies)

For the visual confirmation of the biomolecule’s immobilization after the surface modification process, we incubated two different labeled biomolecules for fluorescent microscopy inspection. After each incubation, samples were washed with DI water and dried under clean air.

**Figure 4:**
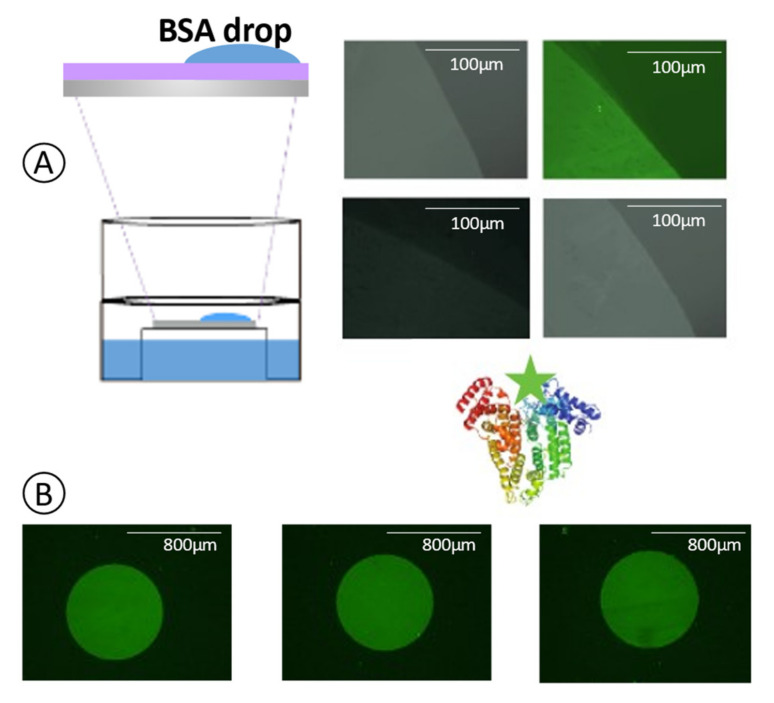
(**A**) Fluorescent microscope image from SiO_2_ biofunctionalization with BSA (**B**) and the labeled antibody on cells A, B, and C of one chip.

Under fluorescent microscope characterization, a clear line was observed differentiating the area of the SiO_2_ chip covered with the drop of BSA by its glowing green color typical from Alexa Fluorophore (Figure 4A). Thus, we confirmed that the surface modification process and the biomolecule immobilization process were successfully achieved. However, BSA is a protein able to bind different types of materials, even through a noncovalent bond, for example, by electrostatic charges where surface modification is not needed. Thus, the challenge here was to immobilize antibodies used as bioreceptors in label-free biological sensors through stable covalent bonds. We applied a PDMS mask within 3 holes (800 µm diameter) on the SiO_2_ chip to define 3 independent sensing areas. In each of them, we incubated the labeled antibodies. For images acquisition, the mask was removed. In Figure 4B, there can be differentiated bright green circles in a black background corresponding to the PDMS mask. The brightness area suggests a successful and uniform antibody immobilization on the incubated area.

### 3.4. Biosensing of MMP9

We evaluated the capacity of anti-MMP9 to recognize specifically the MMP9 protein and demonstrate the robustness and effectiveness of the surface biofunctionalization process applied to label-free optical biosensors.

For that purpose, we selected 3 different SiO_2_ chips to be used as interferometers (Chip 1, Chip 2, Chip 3). We applied the described surface modification protocol and immobilized 50 µg/mL anti-MMP9 on each of them, followed by 1mM ethanolamine surface blocking to prevent fouling events. PDMS masks were then applied to define the 3 independent cells (cell A, cell B, and cell C) on each chip.

**Figure 5:**
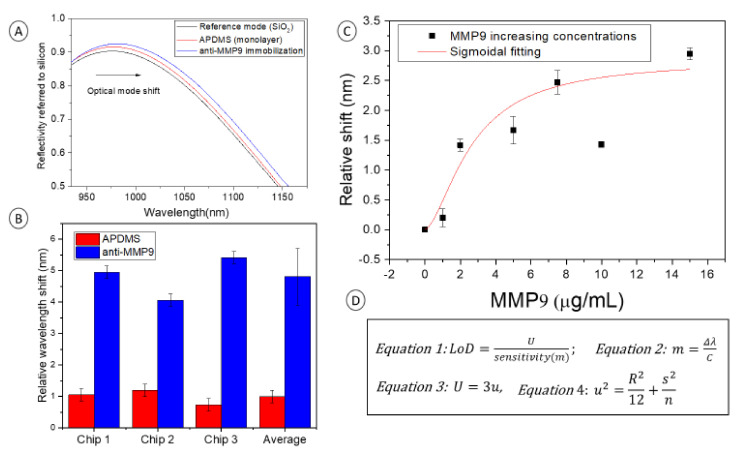
(**A**) Optical mode shift along the wavelength on the different processes: SiO_2_ surface (black), APDMS monolayer (red), and anti-MMP9 immobilization (blue). (**B**) Relative wavelength shift after APDMS surface modification (red columns) and after anti-MMP9 immobilization (blue columns). (**C**) Recognition curve of increasing concentration of MMP9. (**D**) Equations used for the calculations of figure of merit sensitivity and LoD.

In Figure 5A, the optical interferometric response (optical mode) of the reference measurement, the surface modification with APDMS, and the anti-MMP9 immobilization are observed on chip 2. Optical modes are represented by the intensity of the surface reflectance along the wavelength range studied under FT-IR. The shift of the resonant mode in the wavelength of maximum slope around 1075 nm for the different surface modification processes can be noticed with the naked eye.

The red bars in Figure 5B show the shift of the optical mode due to the silane surface modification, which was around 1 nm in the 3 deferent chips. The value from each chip corresponds to the statistical mean of the 3 different cells interrogated defined by the PDMS masks in each chip. Optical mode shifts corresponding to anti-MMP9 immobilization on chips 1, 2, and 3 were 3.9 ± 0.2, 2.8 ± 0.2, and 4.69 ± 0.2 nm, respectively, which is an average of 3.9 ± 0.9 nm. These anti-MMP9 values are referred to as the resonant mode position shift from the APDMS monolayer measurement. The reported values and deviations suggest uniform immobilization of anti-MMP9 along the surface and reproducibility of the chip of the process developed on different chips.

Increasing concentrations of MMP9 (1, 2, 5, 7.5, 10, 15 µg/mL) were incubated on biofunctionalized cells of each chip. The different concentrations were interrogated under FT-IR, showing an optical interferometric response corresponding to different biomolecules’ concentrations. Figure 5C represents the relative shift of the optical mode as a function of the MMP9 concentrations (recognition curve). Shown data correspond to the statistical mean of the 3 interrogated chips, which confers robustness to the shown results. Immobilized anti-MMP9 is considered as the starting point of the recognition curve (concentration 0). A sigmoidal progression of the data is observed, which is commonly seen in biosensors due to the surface saturation after biomolecules recognition. There is an outlier point corresponding to 10 µg/mL, which could be explained by the incubation of a wrong MMP9 concentration solution or to an unlikely systematic error. Obviating the point of concentration 10 µg/mL, the tendency of the point chart follows a sigmoidal fitting (Hill, R-Square 0.93) as is expected in recognition events of increasing concentrations in biosensing processes.

In Figure 5D, the equations used for the sensitivity and the Limit of Detection (LoD) as calculated in other reported works are shown [38]. Although the ambition of this work is not the demonstration of label-free optical biosensor performance, these figures of merit are of high relevance to provide information about biosensing results. Indeed, they verify a successful antibody immobilization to recognize MMP9, specifically.

*LoD* (Equation 2) achieved was 285.71 ng/mL. Equation 3 describes the *sensitivity* (*m*) which was 0.7 nm/µg*mL^−1^, considering the concentration (C) 2 µg/mL as the saturation point where Δ*λ* was 1.41 nm.

(2) $$LoD=\frac{U}{sensitivity\;\left(m\right)}$$

(3) $$m=\frac{\Delta \lambda }{C}$$

(4) U=3u

(5) $$u^{2}=\frac{R^{2}}{12}+\frac{s^{2}}{n}$$

Equations (4) and (5) describe the law of propagation of the uncertainty (*U*) considering a coverage factor of 3. The system resolution (*R*) was 0.01 nm, the standard deviation (*s*) was calculated from the 3 independent cells of each chip. The number of measurements (*n*) was 9, 3 interferometric cells from 3 different chips. The final obtained *U* was 0.2.

These biosensing experiments were developed to show the capacity of anti-MMP9 to detect MMP9 and thus demonstrate the suitability of using the proposed surface modification process for antibodies immobilization in silicon oxide-based biosensors.

Although the proposed sensor is based on simple Fabry–Perot interferometers, the LoD achieved is within the order of other reported label-free biosensors in the literature [39,40] even for the same bioapplication [41]. More complex and sophisticated photonic nanostructures can yield higher theoretical and bulk sensing sensitivities and lower uncertainties. The biofunctionalization protocol proposed can be applied to those nanostructures based on SiO_2_ materials achieving the increased performance to which they were designed.

## 4. Conclusions

Different characterization strategies have been applied in this work to carefully report an effective, uniform, and reproducible biofunctionalization protocol with anti-MMP9 on SiO_2_ surfaces, based on a rarely used silane compound APDMS and CDI linker. We proved by different experimental means that every step described in the process was truly occurring in accordance with the theoretical expectations on each phase of the protocol. Ellipsometer results for the monolayer formation and SCA to notice the change in hydrophilicity along the process showed coherent values for each of the steps. XPS results showed resonant peaks in the expected binding areas for each of the elements and bonds in the different phases. Also, the fluorescent images revealed a standard successful biofunctionalization protocol using labeled BSA and labeled antibodies.

Moreover, MMP9 was detected, confirming that the proposed protocol was not only properly executed but that, indeed, it supposes a relevant endowment for the biosensors researchers and industry. A competitive sensitivity and LoD in the order of ng/mL were obtained.

In addition, this protocol can be applied to other silicon-based optical biosensors as Photonic crystals, Match Zehnder, Ring resonators, which could even be designed and fabricated based on nanostructures (pillars, holes) that enhance the sensitivity of the transducers and the LoD of the biosensor.

Also, the reported process is not exclusive for anti-MMP9 antibodies, in fact, other similar antibodies can be immobilized pursuing other biosensing applications. We have contributed to establishing a common biofunctionalization protocol, and we encourage other researchers to apply it in further biosensors developments.

Finally, all the phases of the biofunctionalization could be developed at a wafer level, increasing the scalability of the process, which could be especially interesting for those willing to fabricate and distribute biosensors at a mass scale.

The finality of this work is to settle down a standard biofunctionalization process in this specific material, on which both the scientific community and incubators and accelerators companies involved in biosensors technology can rely. Outcomes of this study could pave the way for a successful and scalable biofunctionalization process that guides them to final product development.

## Figures and Tables

**Table 1:** Comparison of the structure and monolayer formation of two silanes. APDMS and APTMS.

	APTMS	APDMS
**Chemical Structure**	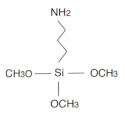	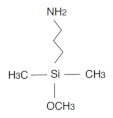
**SCA (°)**	52 ± 1	55 ± 1
**Ellipsometry (nm)**	4.3 ± 0.2	0.6 ± 0.2
**Thickness drawn from XPS (nm)**	3.0	0.5–0.8
**XPS C/N Ratio**	Expected = 3 8.5	Expected = 5 5.6

**Table 2:** Ellipsometry and SCA data from the different biofunctionalization steps.

	Blank SiO_2_	APDMS	APDMS + CDI	APDMS + CDI + BSA	APDMS + CDI + IgG
Thickness (nm)	0 (plasma oxidized chip)	0.7 ± 0.2	0.8 ± 0.2	3.1 ± 0.2	16.7 ± 2
SCA (°)	0 (plasma oxidized chip)	55 ± 1	47 ± 1	75 ± 3	67.1 ± 3
SCA photo	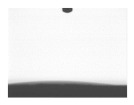	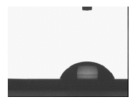	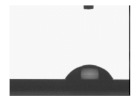	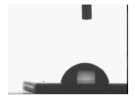	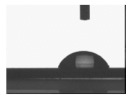

**Table 3:** XPS data of SiO_2_ and thereafter modification with different functional groups (numbers in %).

Surface Atom	SiO_2_	APTMS-SiO_2_	APDMS-SiO_2_	CDI-APDMS-SiO_2_	antiMMP9-CDI-APDMS-SiO_2_
**C 1s**	-	30.6	7.9	19.5	50.9
**Si 2p**	38.0	27.4	35.6	31.8	10.9
**N 1s**	-	3.6	1.4	5.8	9.3
**O 1s**	62.0	38.4	55.1	42.9	28.9
